# Relationship of Handgrip Strength and Body Mass Index With Cognitive Function in Patients With Schizophrenia

**DOI:** 10.3389/fpsyt.2018.00156

**Published:** 2018-04-25

**Authors:** Shinsuke Hidese, Junko Matsuo, Ikki Ishida, Moeko Hiraishi, Toshiya Teraishi, Miho Ota, Kotaro Hattori, Hiroshi Kunugi

**Affiliations:** ^1^Department of Mental Disorder Research, National Center of Neurology and Psychiatry, National Institute of Neuroscience, Tokyo, Japan; ^2^Division of Cognitive and Behavioral Medicine, Department of National Center of Neurology and Psychiatry Brain Physiology and Pathology, Graduate School of Medical and Dental Sciences, Tokyo Medical and Dental University, Tokyo, Japan

**Keywords:** body mass index, cognitive function, handgrip strength, schizophrenia, physical activity

## Abstract

**Background:** The relationship between muscle strength and cognition in schizophrenia has not been well studied. We investigated the potential relationship of handgrip strength (HGS) score and body mass index (BMI) with cognitive function in patients with schizophrenia.

**Methods:** Participants included 153 patients with schizophrenia (age: 36.9 ± 9.4 years; 82 males) and 328 healthy controls (age: 36.4 ± 10.7 years; 150 males), matched for age, sex, and ethnicity (Japanese). HGS was measured using a digital handgrip dynamometer. Cognitive function was evaluated using the Brief Assessment of Cognition in Schizophrenia (BACS) test. A two-way multivariate analysis of covariance was used to compare HGS scores between the patient and control groups. Multiple regression analyses of BACS scores were performed in the patient and control groups using HGS and BMI scores as independent variables.

**Results:** In the intergroup comparison, significantly lower HGS scores were observed in patients with schizophrenia than in healthy controls (*p* < 0.05, corrected). In the patient group, there was a significantly positive correlation between HGS scores and BACS composite score (male, *p* = 0.0014; female, *p* = 0.0051). However, BMI scores were significantly negatively correlated with the BACS composite score (male, *p* = 0.0022; female, *p* = 0.018). Furthermore, the ratio of HGS/BMI was significantly positively correlated with the BACS composite score in the patient group (*p* = 0.00000018).

**Conclusions:** Cognitive function in patients with schizophrenia is correlated positively with HGS and negatively with BMI. HGS/BMI may thus be a good index for cognitive performance in schizophrenia.

## Introduction

Handgrip strength (HGS) is a concise measure used to evaluate muscle strength, and reference values have been published for healthy adults by age and sex [1, 2]. Lower HGS has been associated with sedentary lifestyle [3]. HGS is related to physical and cognitive abilities, particularly in elderly people. This relationship has been demonstrated in systematic reviews reporting that lower HGS is associated with increased risk of cognitive decline in elderly populations [4–7].

Patients with schizophrenia exhibit broad cognitive deficits in the early stages of the illness [8, 9], and physical exercise has been suggested to improve the deficits [10–13]. Light physical activity was positively correlated with cognitive performance [14], while moderate-to-vigorous physical activity was associated with greater cognitive disorganization symptoms in patients with schizophrenia [15]. Although studies regarding HGS remain scarce, lower HGS scores and exacerbated cognitive symptoms were observed in physically inactive patients with schizophrenia [16].

Body mass index (BMI) is a popular index for nutritional status [17] which has been negatively associated with moderate-to-vigorous physical activity [18]. Therefore, BMI may be associated with HGS, which is possibly attributed to physical activity levels. It has been documented that weight gain in schizophrenia can be facilitated by psychotropic medications and unhealthy lifestyle habits [19]. In line with these findings, patients with schizophrenia exhibited more sedentary behavior, which was associated with increased BMI and cognitive symptom scores [20]. In contrast, participation in sports has been demonstrated to reduce BMI and ameliorate psychiatric symptoms in patients with schizophrenia [21].

Obesity (the state of abnormally increased BMI score, BMI ≥ 27.5 kg/m^2^) has been reported to be associated with cognitive impairments in schizophrenia [22]. The MATRICS Consensus Cognitive Battery scores were correlated positively with aerobic fitness (an index of physical ability indicated by VO_2_max) and negatively with increased BMI scores in patients with schizophrenia [23]. Moreover, BMI scores correlated with Eurofit test battery scores, which are assessments of health-related fitness, but not with the HGS subtest score, in patients with schizophrenia or schizoaffective disorder [24]. In addition, [16] reported no correlation between HGS score and cognitive symptoms in a non-elderly sample of patients with schizophrenia (*n* = 80) and healthy adult controls (*n* = 40).

Based on the reported associations between physical and cognitive abilities, it is of particular interest to examine the possible relationship between HGS and cognitive performance in patients with schizophrenia. We aimed to assess this relationship by using the Brief Assessment of Cognition in Schizophrenia (BACS) in a relatively large sample of patients with schizophrenia and healthy controls. It was hypothesized that there would be a positive correlation between HGS score and cognitive function.

## Materials and methods

### Participants

Participants comprised 153 patients with schizophrenia (mean ± standard deviation age: 36.9 ± 9.4 years, 82 males) and 328 healthy controls (36.4 ± 10.7 years, 150 males), matched for age, sex, and ethnicity (Japanese). To exclude the possible effect of aging, only those who were <60 years old were registered. All the participants were self-reported right-handers, and were enrolled through recruitment forms at the National Center of Neurology and Psychiatry, advertisements in free magazines, and our website announcement. The participants were screened for axis I psychiatric disorders by trained psychiatrists using the Japanese version of the Mini-International Neuropsychiatric Interview (M.I.N.I.) [25, 26]. The diagnosis was made in accordance with the Diagnostic and Statistical Manual of Mental Disorders-4th edition criteria [27] based on the information from M.I.N.I. and medical records, if available. All healthy controls were confirmed to have no axis I psychiatric disorders and to have never received psychiatric services. Patients and controls with a medical history of neurological diseases, severe head injury, substance abuse, or mental retardation were excluded. All participants signed consent forms after the study explanation. The study protocol was approved by the ethics committee at the National Center of Neurology and Psychiatry, and applied according to the Declaration of Helsinki [28].

### Clinical and psychological assessments

HGS was measured using a digital handgrip dynamometer (T.K.K.5401; Takei Co., Tokyo, Japan). Right (dominant) and left (non-dominant) HGSs were calculated for each participant by taking the mean of two records conducted for each hand. The average HGS was then calculated for each participant by taking the mean of the dominant and non-dominant HGSs. The severity of symptoms in the patients was evaluated by trained psychiatrists using the Japanese adaptation of the Positive and Negative Syndrome Scale [29, 30]. Cognitive functions were evaluated by trained psychologists using the Japanese version of the BACS [31, 32]. The BACS composite score was the mean *z* score calculated from each BACS score, based on the mean and standard deviation of the healthy controls. Daily doses of antipsychotics were converted to chlorpromazine-equivalent (CPeq) doses according to published guidelines [33].

### Statistical analyses

Continuous and categorical variables were compared between the patient and control groups using Welch's *t-* and chi-square tests, respectively. Correlations for continuous and categorical variables were calculated using Pearson's and Spearman's correlation coefficients, respectively. BACS scores were compared using a one-way (diagnostic) multivariate analysis of covariance (MANCOVA), controlling for age, sex, and BMI. HGS scores were compared using a two-way (diagnosis x sex) MANCOVA, controlling for age and BMI. Comparisons of average HGS among BMI-based classification groups (underweight: BMI < 18.5, normal: 18.5 ≤ BMI < 25, overweight: 25 ≤ BMI < 30, obese: BMI ≥ 30) [34] were conducted using analysis of covariance (ANCOVA), controlling for age and sex. CPeq and any psychotropic medication use were accounted for in the patient group. Multiple regression analyses were performed using the BACS scores as dependent variables and applying independent variables by the forced-entry method. Bonferroni corrections for multiple testing were performed for all the group comparisons, correlations, and multiple regression analyses. For instance, as correlations and multiple regression analyses were repeated with regard to all seven BACS scores, the statistical significance level was set to *p* < 0.05/7. Effect sizes were calculated by Cohen's d for the *t*-test, ϕ for the chi-square test, η^2^ for the ANCOVA and MANCOVA, and adjusted *R*^2^ for the multiple regression analysis. Statistical analyses were conducted using the Statistical Package for the Social Sciences version 24.0 (SPSS Japan, Tokyo, Japan). All statistical tests were two-tailed, and a *p*-value of <0.05 was deemed significant.

## Results

### Analyses of clinical variables

Demographic and clinical characteristics of the participants are depicted in Table 1. Mean BMI was significantly higher in patients with schizophrenia than in healthy controls after correction for multiple testing. With regard to BMI-based classification, the proportion of normal participants was significantly lower (odds ratio = 0.45, 95% confidence interval: 0.30–0.68), while that of obese participants was significantly higher in the patient group than in the control group (odds ratio = 4.52, 95% confidence interval: 2.00–10.40; corrected). A MANCOVA analysis revealed that all of the BACS scores were significantly lower in the patient group than in the control group (corrected).

**Table 1:** Demographic and clinical characteristics of the participants.

	**Patients with schizophrenia (*****n*** = **153)**		**Healthy controls (*****n*** = **328)**		
	**Mean ± Standard deviation**	**Range**	**Mean ± Standard deviation**	**Range**	**Statistical comparison**
Age (years)	36.9 ± 9.4	18-58	36.4 ± 10.7	18–59	*t*_(334.7)_ = −0.49, *p* = 0.63, Cohen's d = 0.05
Sex, male (%)	82 (53.6)		150 (45.7)		$\chi_{\left(1\right)}^{2}$ = 2.58, *p* = 0.11, ϕ = −0.07
Education (years)	14.0 ± 2.4	9–22	15.1 ± 2.1	9–22	*t*_(267.1)_ = 5.02, ***p*** = **9.4.E-07**, Cohen's d = 0.51
Body mass idex (kg/m^2^)	24.3 ± 5.1	14.3–45.2	22.1 ± 3.4	15.8–34.3	*t*_(209.5)_ = −4.68, ***p*** = **5.0.E-06**, Cohen's d = 0.54
Underweight	15 (9.8)		26 (7.9)		$\chi_{\left(1\right)}^{2}$ = 0.55, *p* = 0.46, ϕ = 0.03
Normal	85 (55.5)		43 (74.1)		$\chi_{\left(1\right)}^{2}$ = 15.05, ***p*** = **1.0.E-04**, ϕ = −0.18 OR = 0.45, 95%CI:0.30-0.68
Overweight	32 (20.9)		47 (14.3)		$\chi_{\left(1\right)}^{2}$ = 3.62, *p* = 0.057, ϕ = 0.09
Obese	17 (11.1)		9 (2.7)		$\chi_{\left(1\right)}^{2}$ = 14.71, ***p*** = **1.3.E-04**, ϕ = 0.18 OR = 4.52, 95%CI:2.00-10.40
**BACS**					
Verbal memory	40.6 ± 12.8	10–71	51.5 ± 9.6	20–75	*F*_(1, 468)_ = 89.34, ***p*** = **1.6.E-19**, η^2^ = 0.13
Working memory	18.7 ± 4.5	8–28	22.1 ± 3.7	10–28	*F*_(1, 468)_ = 61.50, ***p*** = **3.0.E-14**, η^2^ = 0.10
Motor speed	70.7 ± 16.4	22–100	83.4 ± 12.1	40–100	*F*_(1, 468)_ = 70.17, ***p*** = **6.4.E-16**, η^2^ = 0.12
Verbal fluency	43.5 ± 12.3	14–76	51.8 ± 10.3	26–87	*F*_(1, 468)_ = 41.10, ***p*** = **3.5.E-10**, η^2^ = 0.08
Attention	54.4 ± 13.3	19–86	72.1 ± 12.9	37–100	*F*_(1, 468)_ = 174.18, ***p*** = **4.9.E-34**, η^2^ = 0.22
Executive function	16.7 ± 3.4	4–22	18.3 ± 2.6	3–22	*F*_(1, 468)_ = 26.64, ***p*** = **3.6.E-07**, η^2^ = 0.05
Composite	−1.00 ± 0.9	−3.89–1.01	0.00 ± 0.6	−1.94–1.50	*F*_(1, 468)_ = 168.50, ***p*** = **4.0.E-33**, η^2^ = 0.22
Age of onset (years)	23.1 ± 7.2	5–54			
Duration of illness (years)	13.7 ± 8.9	1–38			
**CPeq (mg/day)**					
Typical antipsychotics	95.8 ± 220.7	0–1362.5			
Atypical antipsychotics	349.3 ± 375.7	0–1860			
Total antipsychotics	445.2 ± 410.3	0–1960			
Antiparkinson medication use (%)	57 (37.3)				
Minor tranquilizer use (%)	89 (58.2)				
Any psychotropic medication use (%)	131 (85.6)				
**PANSS**					
Positive	13.6 ± 5.0	7–32			
Negative	15.8 ± 6.1	7–33			
General psychopathology	29.9 ± 8.3	16–53			

*BACS, Brief Assessment of Cognition in Schizophrenia; CI, confidence interval; CPeq, chlorpromazine-equivalent dose; OR, odds ratio; PANSS, Positive and Negative Syndrome Scale*.

*Significant p-values (p < 0.016 for the t-test, p < 0.01 for the chi-square test, and p < 0.007 for the multivariate analysis of covariance, corrected for multiple testing) are shown in bold exponents. There were 4 and 3 missing values of BMI scores in the patients and controls, respectively*.

### Analyses of HGS scores

HGS scores of the participants are depicted in Table 2. A two-way MANCOVA revealed that all HGS scores were significantly lower in the patient group than in the control group, and were significantly higher in males than in females (corrected). The average HGS (i.e., the average of left and right HGS scores) was used as “the HGS score” in the following analyses. Correlations between HGS score and clinical variables are demonstrated in Table S1. Of note, BMI was significantly positively correlated with HGS score in both groups (patient: *r* = 0.27, *p* = 8.0.E-04; control: *r* = 0.40, *p* = 3.4.E-14). Comparisons of HGS scores by BMI-based classification are displayed in Figure S1.

**Table 2:** Handgrip strength scores of the participants.

	**Male**				**Female**				
	**Patient (*****n*** = **82)**		**Control (*****n*** = **150)**		**Patient (*****n*** = **71)**		**Control (*****n*** = **178)**		**Statistical comparison**
	**Mean ± SD**	**Range**	**Mean ± SD**	**Range**	**Mean ± SD**	**Range**	**Mean ± SD**	**Range**	**For diagnosis**
Average hangrip strength (kg)	35.9 ± 7.4	16.4–52.0	38.0 ± 6.1	22.6–52.2	22.8 ± 4.7	9.5–41.9	24.9 ± 4.3	13.8–39.2	*F*_(1, 468)_ = 27.0, ***p*** = **3.0.E-07**, η^2^ = 0.023
Right handgrip strength (kg)	37.0 ± 7.6	18.1–55.2	38.9 ± 6.4	24.7–55.0	23.2 ± 5.4	8.0–45.3	25.7 ± 4.6	10.9–41.7	*F*_(1, 468)_ = 24.2, ***p*** = **1.7.E-06**, η^2^ = 0.022
Left handgrip strength (kg)	34.8 ± 7.7	13.0–48.8	37.1 ± 6.3	20.5–51.8	22.3 ± 4.4	10.9–38.6	24.2 ± 4.4	12.5–36.8	*F*_(1, 468)_ = 25.2, ***p*** = **7.2.E-08**, η^2^ = 0.021

*SD, standard deviation. Significant p-values (p < 0.016; corrected for multiple testing) are shown in bold exponents*.

*There was no significant interaction between diagnosis and sex (p = 0.64 for the average, p = 0.35 for the right, and p = 0.96 for the left handgrip strength scores, respectively)*.

### Correlation of HGS and BMI scores with cognitive function

Multiple regression analysis for each sex involving BACS scores and forced-entry variables are documented in Table 3. BACS scores were used as dependent variables, while HGS scores, age, BMI, education (years), CPeq (for patients), and any psychotropic medication use (for patients) were used as independent variables. Attention (β = 0.47, *p* = 4.0.E-05) and composite (β = 0.36, *p* = 1.4.E-03) scores were significantly positively correlated with HGS scores in male patients (corrected). Motor speed (β = 0.34, *p* = 1.5.E-03), attention (β = 0.36, *p* = 1.4.E-03), and composite (β = 0.30, *p* = 5.1.E-03) scores were significantly positively correlated with HGS scores in female patients (corrected). Motor speed (β = −0.40, *p* = 1.3.E-03) and composite (β = −0.36, *p* = 2.2.E-03) scores were significantly negatively correlated with BMI in male patients (corrected). Working memory was significantly negatively correlated with BMI in female patients (β = −0.34, *p* = 6.9.E-03, corrected). No significant correlations were observed between the HGS and BACS scores in male or female controls. However, working memory (β = −0.21, *p* = 5.8.E-03) and composite (β = −0.22, *p* = 2.8.E-03) scores were significantly negatively correlated with BMI in female controls (corrected).

**Table 3:** Multiple regression analysis for each sex involving Brief Assessment of Cognition in Schizophrenia (BACS) scores and forced-entry variables.

	**Verbal memory**		**Working memory**		**Motor speed**		**Verbal fluency**		**Attention**		**Executive function**		**Composite**	
	β	***p***	β	***p***	β	***p***	β	***p***	β	***p***	β	***p***	β	***p***
**<Male patients**>	*R*^2^ = 0.09		*R*^2^ = 0.08		*R*^2^ = 0.16		*R*^2^ = 0.18		*R*^2^ = 0.29		*R*^2^ = 0.003		*R*^2^ = 0.27	
Handgrip strength (kg)	0.15	0.21	0.16	0.19	0.29	0.01	0.23	0.05	0.47	**4.0E-05**	0.10	0.44	0.36	**1.4E-03**
Age (years)	−0.13	0.29	−0.10	0.41	0.10	0.41	0.11	0.35	−0.14	0.21	0.08	0.51	−0.02	0.86
Education (years)	0.15	0.22	0.15	0.23	−0.04	0.72	−0.05	0.68	0.08	0.48	0.02	0.87	0.08	0.47
Body mass idex (kg/m^2^)	−0.22	0.09	−0.23	0.07	−0.40	**1.3.E-03**	−0.29	0.02	−0.26	0.02	−0.11	0.39	−0.36	**2.2E-03**
CPeq total antipsychotics (mg/day)	−0.11	0.36	−0.05	0.67	−0.19	0.12	0.01	0.91	−0.07	0.50	0.10	0.42	−0.07	0.50
Any psychotropic medication use (%)	−0.09	0.48	−0.11	0.40	0.09	0.47	−0.34	**5.9.E-03**	−0.14	0.20	−0.26	0.05	−0.21	0.06
**<Female patients**>	*R*^2^ = 0.25		*R*^2^ = 0.22		*R*^2^ = 0.35		*R*^2^ = 0.13		*R*^2^ = 0.30		*R*^2^ = 0.16		*R*^2^ = 0.37	
Handgrip strength (kg)	0.16	0.16	0.10	0.36	0.34	**1.5E-03**	0.25	0.04	0.36	**1.4E-03**	0.16	0.18	0.30	**5.1.E-03**
Age (years)	−0.35	**2.5.E-03**	−0.19	0.10	0.05	0.61	−0.06	0.60	−0.14	0.19	−0.19	0.11	−0.20	0.05
Education (years)	0.26	0.03	0.17	0.16	0.21	0.05	0.25	0.04	0.14	0.21	0.00	0.98	0.22	0.04
Body mass idex (kg/m^2^)	−0.16	0.17	−0.34	**6.9.E-03**	−0.15	0.18	−0.25	0.05	−0.29	0.01	−0.05	0.69	−0.26	0.02
CPeq total antipsychotics (mg/day)	−0.09	0.45	−0.17	0.18	−0.17	0.14	0.02	0.89	−0.22	0.07	−0.37	**6.9.E-03**	−0.23	0.05
Any psychotropic medication use (%)	−0.11	0.38	−0.05	0.73	−0.32	0.01	−0.06	0.68	−0.06	0.65	−0.03	0.82	−0.14	0.25
**<Male controls**>	*R*^2^ = 0.24		*R*^2^ = 0.24		*R*^2^ = 0.13		*R*^2^ = 0.08		*R*^2^ = 0.32		*R*^2^ = 0.04		*R*^2^ = 0.28	
Handgrip strength (kg)	0.01	0.89	0.01	0.89	0.03	0.70	−0.01	0.89	−0.01	0.93	0.12	0.14	0.04	0.63
Age (years)	−0.46	**3.9.E-08**	−0.46	**3.9.E-08**	−0.23	0.01	−0.06	0.48	−0.49	**1.0.E-09**	−0.16	0.08	−0.36	**6.4.E-06**
Education (years)	−0.11	0.16	0.03	0.72	0.19	0.01	0.24	**3.5.E-03**	0.16	0.02	0.13	0.11	0.27	**2.9E-04**
Body mass idex (kg/m^2^)	0.03	0.72	−0.11	0.16	−0.16	0.07	−0.08	0.37	−0.09	0.23	−0.03	0.71	−0.15	0.06
**<Female controls**>	*R*^2^ = 0.10		*R*^2^ = 0.08		*R*^2^ = 0.00		*R*^2^ = 0.08		*R*^2^ = 0.17		*R*^2^ = 0.05		*R*^2^ = 0.19	
Handgrip strength (kg)	0.04	0.60	0.04	0.58	0.07	0.39	0.14	0.06	0.15	0.04	0.06	0.46	0.14	0.06
Age (years)	−0.23	**4.8.E-03**	−0.10	0.23	0.00	0.97	0.10	0.25	−0.20	0.01	−0.02	0.77	−0.12	0.11
Education (years)	0.17	0.04	0.14	0.08	0.01	0.88	0.27	**9.3.E-04**	0.24	**2.2.E-03**	0.23	**5.4.E-03**	0.29	**1.5E-04**
Body mass idex (kg/m^2^)	−0.06	0.44	−0.21	**5.8.E-03**	−0.15	0.06	−0.12	0.11	−0.17	0.02	−0.07	0.38	−0.22	**2.8.E-03**

*CPeq, chlorpromazine-equivalent dose. Significant p-values (p < 0.0071; corrected for multiple testing) are shown in bold exponents*.

Based on these results, further multiple regression analysis was conducted using a newly-defined index, namely the ratio of HGS to BMI (Table 4). BACS scores were used as dependent variables, while HGS/BMI, age, sex, education (years), CPeq (for patients), and any psychotropic medication use (for patients), were used as independent variables. In this case, working memory (β = 0.35, *p* = 9.7.E-04), motor speed (β = 0.47, *p* = 1.2.E-05), verbal fluency (β = 0.40, *p* = 2.3.E-04), attention (β = 0.56, *p* = 2.9.E-08), and composite (β = 0.51, *p* = 1.8.E-07) scores were significantly positively correlated with HGS/BMI in the patient group (corrected). Composite score was significantly positively correlated with HGS/BMI in the control group (β = 0.20, *p* = 2.6.E-03, corrected).

**Table 4:** Multiple regression analysis between Brief Assessment of Cognition in Schizophrenia (BACS) scores and handgrip strength/body mass index.

	**Verbal memory**		**Working memory**		**Motor speed**		**Verbal fluency**		**Attention**		**Executive function**		**Composite**	
	**β**	***p***	**β**	***p***	**β**	***p***	**β**	***p***	**β**	***p***	**β**	***p***	**β**	***p***
**<Patients with schizophrenia**>	*R*^2^ = 0.19		*R*^2^ = 0.19		*R*^2^ = 0.23		*R*^2^ = 0.15		*R*^2^ = 0.32		*R*^2^ = 0.09		*R*^2^ = 0.35	
Handgrip strength/body mass index (m^2^)	0.26	0.01	0.35	**9.7.E-04**	0.47	**1.2E-05**	0.40	**2.3.E-04**	0.56	**2.9E-08**	0.15	0.17	0.51	**1.8E-07**
Age (years)	−0.26	**1.7.E-03**	−0.14	0.08	0.06	0.47	0.01	0.91	−0.14	0.06	−0.07	0.39	−0.13	0.08
Sex	0.19	0.07	0.06	0.53	0.38	**2.0.E-04**	0.21	0.05	0.37	**9.4E-05**	−0.11	0.29	0.26	**4.6.E-03**
Education (years)	0.22	0.01	0.17	0.04	0.07	0.36	0.10	0.21	0.12	0.12	0.04	0.65	0.17	0.02
CPeq total antipsychotics (mg/day)	−0.10	0.24	−0.08	0.33	−0.17	0.04	0.01	0.91	−0.13	0.10	−0.10	0.27	−0.13	0.09
Any psychotropic medication use (%)	−0.10	0.25	−0.10	0.25	−0.09	0.30	−0.18	0.04	−0.07	0.37	−0.15	0.10	−0.16	0.04
**<Healthy controls**>	*R*^2^ = 0.19		*R*^2^ = 0.09		*R*^2^ = 0.02		*R*^2^ = 0.08		*R*^2^ = 0.25		*R*^2^ = 0.06		*R*^2^ = 0.22	
Handgrip strength / body mass index (m^2^)	0.10	0.15	0.14	0.04	0.10	0.19	0.13	0.08	0.17	0.01	0.12	0.10	0.20	**2.6.E-03**
Age (years)	−0.37	**5.7.E-12**	−0.20	**3.7.E-04**	0.00	0.98	0.00	0.93	−0.37	**9.9.E-13**	−0.09	0.12	−0.28	**1.3.E-07**
Sex	0.25	**2.3.E-04**	0.07	0.35	0.10	0.19	0.24	**1.1.E-03**	0.27	**4.5.E-05**	0.02	0.78	0.25	**1.7.E-04**
Education (years)	0.08	0.12	0.16	**4.8.E-03**	0.15	0.01	0.25	**1.2.E-05**	0.18	**3.1.E-04**	0.18	**1.6.E-03**	0.27	**3.7.E-07**

*CPeq, chlorpromazine-equivalent dose. Significant p-values (p < 0.0071; corrected for multiple testing) are shown in bold exponents. Sex was coded as male: 1 and female: 2*.

### Correlation between cognitive function and clinical variables

**Figure 1:**
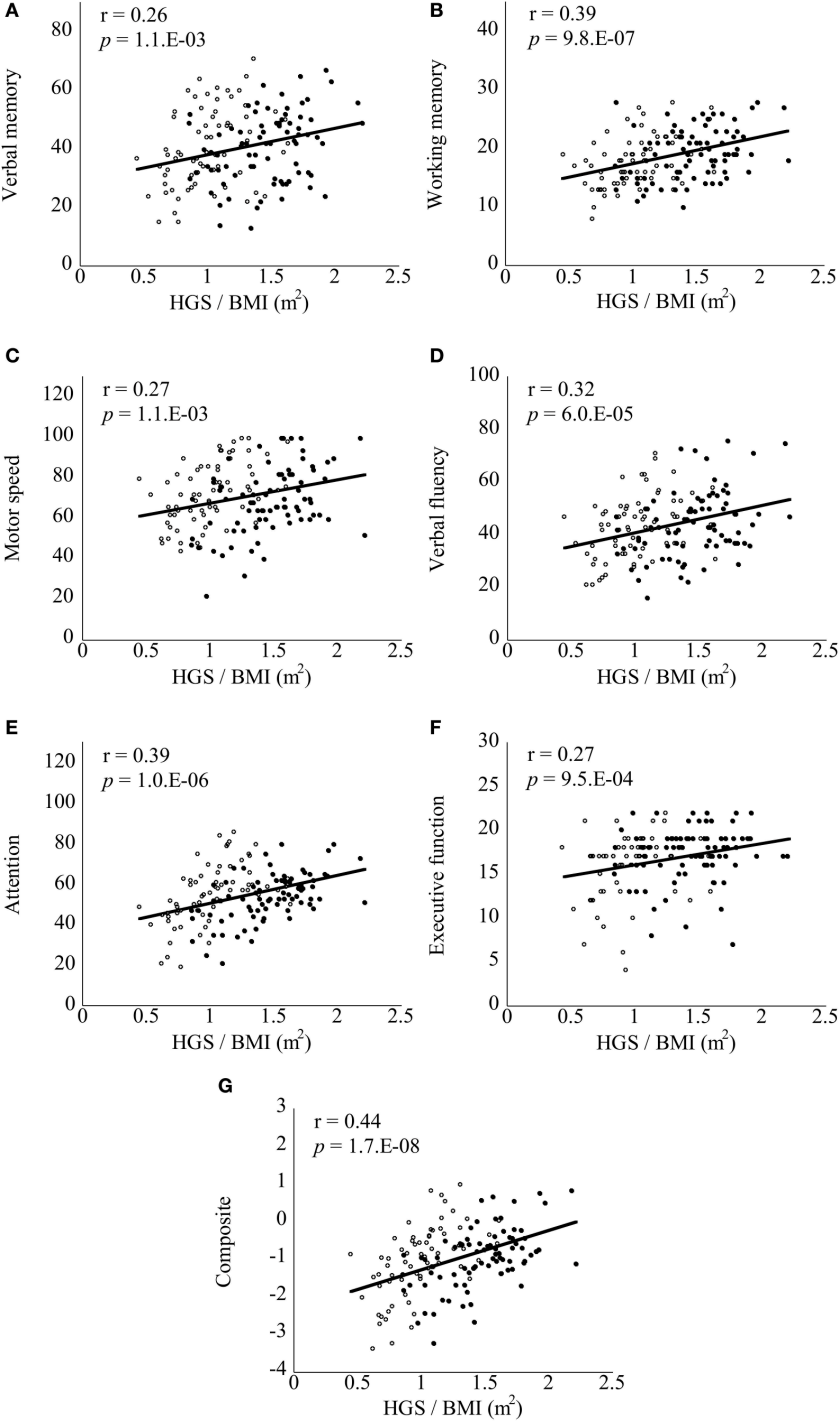
Scatter plots of correlations between handgrip strength (HGS)/body mass index (BMI) and Brief Assessment of Cognition in Schizophrenia (BACS) scores in patients with schizophrenia. HGS/BMI positively correlated with all of the BACS scores **(A–G)** in the patients with schizophrenia (all *p* < 0.0071, corrected for multiple testing). Black and white circles indicate male and female patients, respectively. Significant *p*-values are shown in exponents. r, Pearson's correlation coefficient.

**Figure 2:**
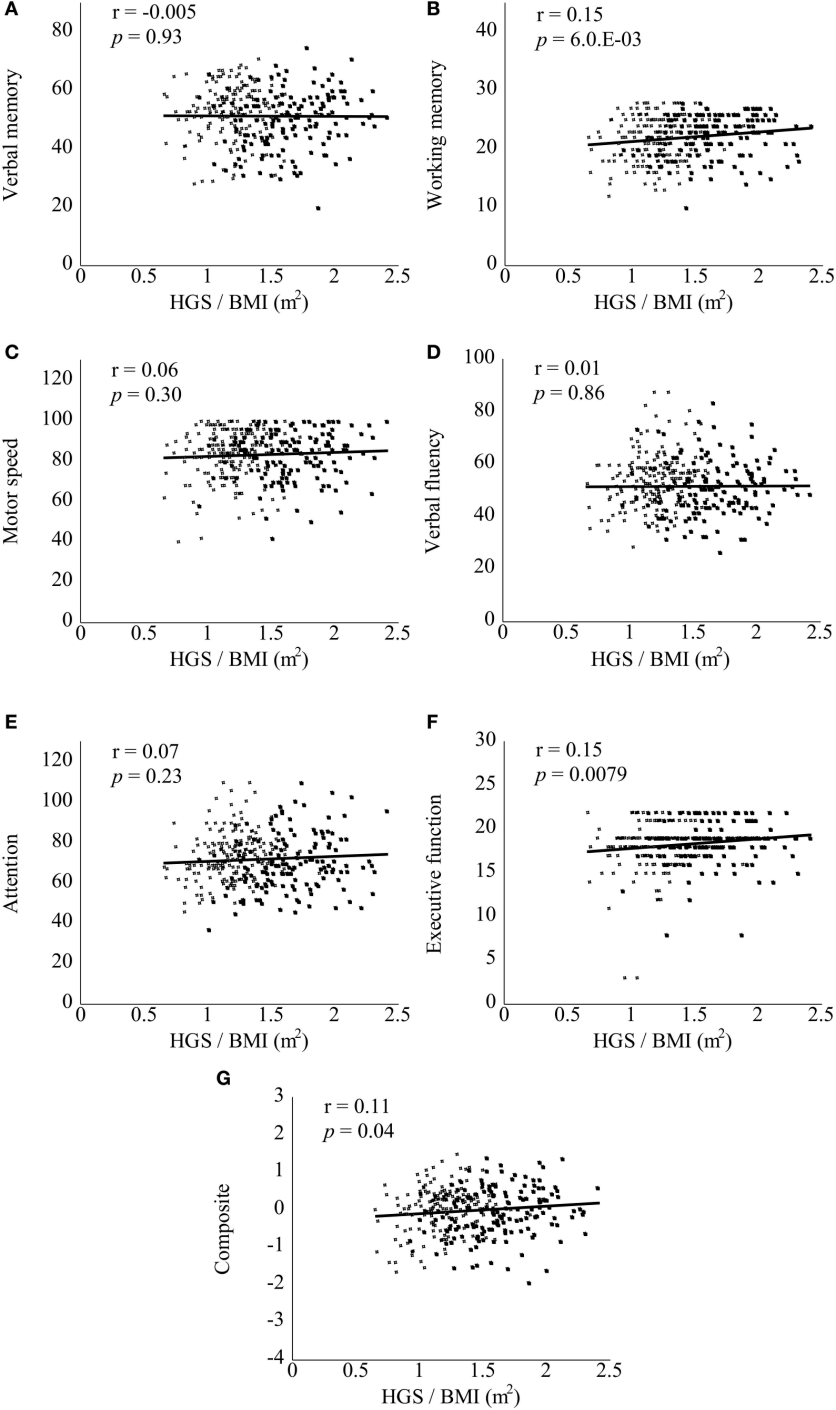
Scatter plots of correlations between handgrip strength (HGS)/body mass index (BMI) and Brief Assessment of Cognition in Schizophrenia (BACS) scores in healthy controls. HGS/BMI positively correlated with only the BACS working memory score (**B**, not **A** or **C–G**) in the controls (*p* < 0.0071, corrected for multiple testing). Black and white squares indicate male and female controls, respectively. A significant *p*-value is shown in an exponent. r, Pearson's correlation coefficient.

Correlations between the BACS scores and clinical variables are documented in Table S2. In bivariate correlation analyses, attention (*r* = 0.23, *p* = 3.5.E-03) and composite (*r* = 0.25, *p* = 2.3.E-03) scores were significantly positively correlated with HGS score in the patient group (corrected). Verbal memory (*r* = −0.26, *p* = 1.5.E-03), working memory (*r* = −0.30, *p* = 2.2.E-04), motor speed (*r* = −0.28, *p* = 6.1.E-04), verbal fluency (*r* = −0.26, *p* = 1.1.E-03), attention (*r* = −0.27, *p* = 7.8.E-04), and composite (*r* = −0.33, *p* = 4.4.E-05) scores were significantly negatively correlated with BMI in the patient group (corrected). Notably, all of the BACS scores were significantly positively correlated with HGS/BMI in the patient group (Figure 1, corrected). In the control group, BACS scores were not significantly correlated with HGS score. However; verbal memory (*r* = −0.24, *p* = 7.9.E-06), working memory (*r* = −0.21, *p* = 1.6.E-04), attention (*r* = −0.27, *p* = 5.5.E-07), and composite (*r* = −0.28, *p* = 4.3.E-07) scores were significantly negatively correlated with BMI in the control group (corrected). By contrast, only working memory score showed a significantly positive correlation with HGS/BMI in the control group (Figure 2, corrected).

## Discussion

The present study examined the association of HGS and BMI with cognitive function. HGS scores were significantly reduced in patients with schizophrenia compared to healthy controls. The HGS and BMI scores and the HGS/BMI were significantly associated with cognitive impairments in the patient group. However, the HGS/BMI was significantly associated with only the BACS composite score in the control group. Our findings suggest that HGS and BMI have positive and negative relationships, respectively, with cognitive function in both male and female patients with schizophrenia.

We found that patients with schizophrenia obtained lower HGS scores than in healthy controls, irrespective of sex or BMI. The HGS scores exhibited a positive correlation with the BACS motor speed, attention, and composite scores in the patient group. A previous study reported no correlation between HGS and cognitive scores measured by the psychosis evaluation tool for common use by caregivers [16]. In contrast, the current study demonstrates for the first time several correlations between HGS and cognitive measures using a comprehensive cognitive battery. Our results suggest a positive relationship between HGS and cognitive function in patients with schizophrenia under the age of 60 years. As reported with regard to elderly cognitive decline [4–7], HGS may be used as an additional index to reflect cognitive deficits in schizophrenia, particularly deficits of motor speed and attention. These deficits may be influenced by physical activity levels [14, 15].

BMI was negatively correlated with the BACS working memory, motor speed, and composite scores in the patient group. These correlations agree with prior findings [22, 23], and with our recent study reporting that obesity is associated with poorer cognitive function in patients with major depressive disorder [35]. Increased BMI in schizophrenia may be associated with lower physical activity levels [20, 23], although our data did not take physical activity into account. In addition, we observed a positive correlation between BMI and HGS scores in both the patient and control groups. These findings are in contrast with previous findings in patients with schizophrenia or schizoaffective disorder [24]. However, similar results to ours have been obtained in healthy controls [2, 36]. In addition, our BMI-based comparisons showed that higher and lower HGS scores were observed in obese and underweight participants, respectively. Taking the relationships of both HGS and BMI scores into consideration, the optimal BMI range (i.e., normal) may be beneficial for cognitive function in schizophrenia.

The ratio of HGS/BMI is a newly-defined index based on the former regression analyses. This ratio positively correlated with the BACS working memory, motor speed, verbal fluency, attention, and composite scores in the patient group. Furthermore, in bivariate correlation analyses, the ratio positively correlated with all BACS scores in the patients. These results suggest that the HGS/BMI could be a concise and useful index to estimate the level of cognitive performance in schizophrenia. In this context, higher HGS and lower BMI scores may contribute to the promotion of schizophrenic cognitive function. With regard to treatment, interventions for HGS and BMI using physical exercise have the potential to ameliorate cognitive dysfunctions in patients with schizophrenia. Such effects have previously been reported in cases of dementia [37, 38] and schizophrenia [39–42]. Considering the positive correlation between HGS and BMI scores, it is highly plausible that the balance of high HGS and low BMI scores, namely a higher HGS relative to BMI may be preferable for cognitive performance in schizophrenia.

Multiple regression analyses revealed that there were no effects of HGS on cognitive function, while the HGS/BMI was positively correlated with only the composite score in healthy controls. Notably, the HGS/BMI was positively associated with cognitive function in healthy controls, although the associations appeared to be weaker compared to patients with schizophrenia. Ceiling effects may contribute to the weaker association, as HGS and BACS scores were higher and BMI scores were lower in controls compared to patients. Moreover, specialized interactions between physical ability and cognitive function may be present in patients with schizophrenia, as suggested by previous studies [10–13]. Lifestyle factors observed in patients with schizophrenia [19], such as lower physical activity levels and higher BMI, may have influenced the difference between the patients and controls. Indeed, patients with schizophrenia were reported to be physically inactive compared to healthy controls [16], although data related to physical activity were not included in the present study.

This study had several limitations. First, the majority of the patients (85.7%) had taken psychotropic medications. These effects were accounted for by the ANCOVA and multiple regression analyses. Second, we conducted corrections for multiple testing using the Bonferroni method which may have resulted in type II errors due to the conservative nature of the analysis. Lastly, the cross-sectional approach of the current study cannot draw any conclusions concerning causal relationships. Given the correlational nature of the analysis, we cannot clarify whether cognitive dysfunction is the cause or result of low HGS in schizophrenia. It is possible that physical disabilities and cognitive deficits have individual and negative impacts on daily functioning in schizophrenia. This theory is supported by the therapeutic effects of aerobic exercise on brain structure and function via neuroplastic changes in patients with schizophrenia [43–46].

In conclusion, patients with schizophrenia showed lower HGS scores compared to healthy controls. In the patient group, HGS and BMI scores correlated positively and negatively with cognitive functions, respectively. Furthermore, the ratio of HGS/BMI positively correlated with the majority of the cognitive functions examined in the patient group. HGS/BMI may thus be a good index for cognitive performance in schizophrenia. These results suggest that a close relationship exists between physical status (muscle strength and BMI) and cognitive function in patients with schizophrenia.

## Authors contributions

SH designed, and HK supervised the study; JM, II, and MH assessed cognitive function by the BACS. SH, TT, MO, and KH determined psychiatric diagnoses and evaluated symptoms by the Positive and Negative Syndrome Scale; SH performed the statistical analysis and wrote the draft of the manuscript. All authors have approved the final manuscript.

### Conflict of interest statement

The authors declare that the research was conducted in the absence of any commercial or financial relationships that could be construed as a potential conflict of interest.

## Abbreviations

ANCOVA: analysis of covariance
BACS: Brief Assessment of Cognition in Schizophrenia
BMI: body mass index
CPeq: chlorpromazine-equivalent
HGS: handgrip strength
MANCOVA: multivariate analysis of covariance
M.I.N.I.: mini-international neuropsychiatric interview.
