# Supplementary material for: Relationship of Handgrip Strength and Body Mass Index With Cognitive Function in Patients With Schizophrenia
Source: Front Psychiatry. 2018 Apr 25;9:156. doi: 10.3389/fpsyt.2018.00156 (PMC5930849; doi:10.3389/fpsyt.2018.00156)
Supplement: Supplementary file 1 [file Image_1.pdf]

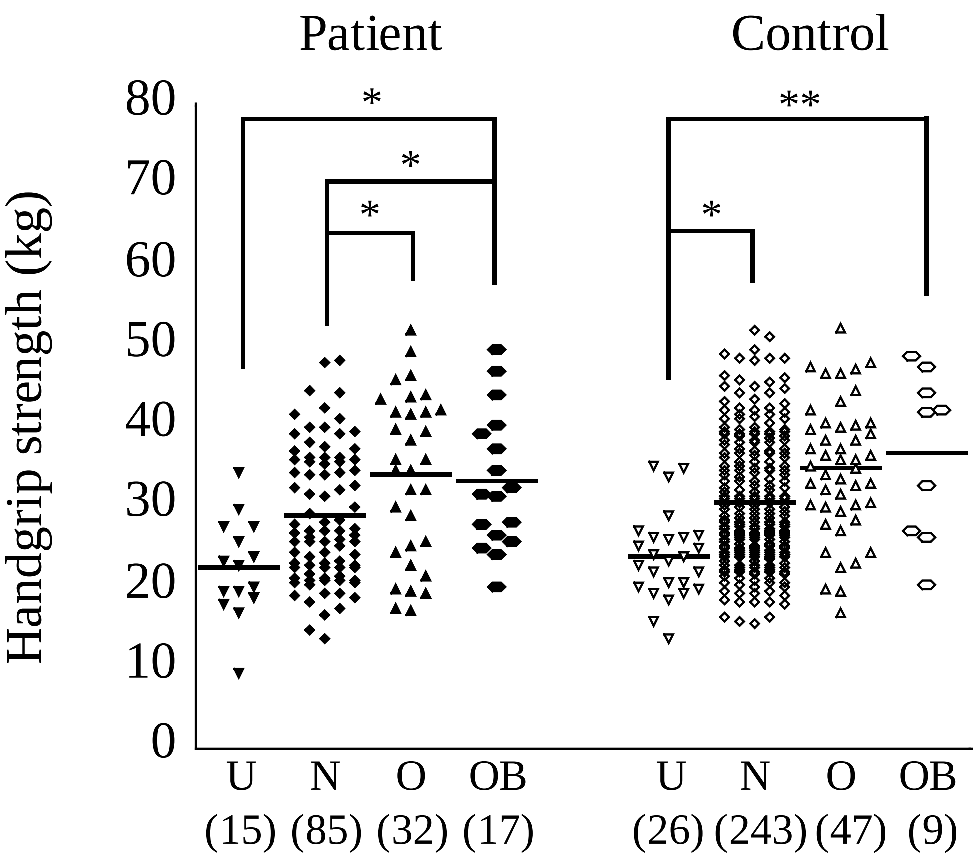

Figure S1. Comparisons of handgrip strength scores by body mass index-based classification.

In patients with schizophrenia, significantly lower scores were observed in the underweight group compared to obese group (corrected $p = 0.033$). In addition, significantly lower scores were observed in the normal group compared to overweight (corrected $p = 0.023$) and obese (corrected $p = 0.022$) groups (F [3, 137] = 5.59, $\eta^2 = 0.049$). In healthy controls, significantly lower scores were observed in the underweight group compared to normal (corrected $p = 0.023$) and obese (corrected $p = 0.002$) groups (F [3, 319] = 5.04, $\eta^2 = 0.018$). Horizontal lines in dots indicate the mean scores. Numbers in parentheses represent the numbers of participants. $^{*}p < 0.05$, $^{**}p < 0.01$.

U, underweight; N, normal; O, overweight; OB, obese
